# Improvement of Crystal Quality of AlN Films with Different Polarities by Annealing at High Temperature

**DOI:** 10.3390/mi13010129

**Published:** 2022-01-14

**Authors:** Yang Yue, Maosong Sun, Jie Chen, Xuejun Yan, Zhuokun He, Jicai Zhang, Wenhong Sun

**Affiliations:** 1 Research Center for Optoelectronic Materials and Devices, School of Physical Science and Technology, Guangxi University, Nanning 530004, China; 1907301107@st.gxu.edu.cn (Y.Y.); 1907401011@st.gxu.edu.cn (M.S.); a709161389@163.com (J.C.); xjuny100@163.com (X.Y.); hzkgmy@hotmail.com (Z.H.); 2 College of Mathematics and Physics, Beijing University of Chemical Technology, Beijing 100029, China; 3 State Key Laboratory of Chemical Resource Engineering, Beijing University of Chemical Technology, Beijing 100029, China; 4 Guangxi Key Laboratory of Processing for Non-Ferrous Metal and Featured Materials, Guangxi University, Nanning 530004, China; 5 Guangxi Key Laboratory for Relativistic Astrophysics, School of Physical Science & Technology, Guangxi University, Nanning 530004, China

**Keywords:** AlN, polar, semi-polar, non-polar, magnetron sputtering, HTA

## Abstract

High-quality AlN film is a key factor affecting the performance of deep-ultraviolet optoelectronic devices. In this work, high-temperature annealing technology in a nitrogen atmosphere was used to improve the quality of AlN films with different polarities grown by magnetron sputtering. After annealing at 1400–1650 °C, the crystal quality of the AlN films was improved. However, there was a gap between the quality of non-polar and polar films. In addition, compared with the semi-polar film, the quality of the non-polar film was more easily improved by annealing. The anisotropy of both the semi-polar and non-polar films decreased with increasing annealing temperature. The results of Raman spectroscopy, scanning electron microscopy and X-ray photoelectron spectroscopy revealed that the annihilation of impurities and grain boundaries during the annealing process were responsible for the improvement of crystal quality and the differences between the films with different polarities.

## 1. Introduction

The III-nitride semiconductor materials are a popular research topic because of their wide applications in visible and ultraviolet light-emitting diodes, laser diodes and high-power electronic devices [1,2,3,4]. Among them is AlN, a promising material with many advantages such as high thermal conductivity, high temperature resistance and stable chemical properties [5,6,7,8]. On account of the limitations of wafer size and the production cost, AlN materials are currently mostly epitaxial films grown on sapphire substrates [9,10,11]. However, the lattice and thermal mismatch between the AlN film and the substrate will result in a high dislocation density in the epilayer [12]. High-quality AlN materials are extremely important for improving the related device performance. Additionally, when the material is grown along a [0001] direction, the large polarization field influences its properties significantly [13,14,15]. Semi-polar and non-polar materials are usually used to avoid the influence of the built-in electric field. Yusuke Yoshizumi et al. fabricated high-performance green laser diodes by using a semi-polar thin film [16]. Hwang, S.M. et al. [17] prepared non-polar a-plane light-emitting diodes (LED) on an r-plane sapphire substrate and proved that the crystallization quality of non-polar materials strongly affects the device performance. The preparation of high-quality semi-polar and non-polar materials is extremely important. However, due to the anisotropy of the atomic structure on the surface of semi-polar and non-polar thin films, there are a lot of dislocations and stacking faults formed during growth [18,19]. These defects make the quality of semi-polar and non-polar AlN thin films much lower than the quality of the polar AlN films and limit the practical application of semi-polar and non-polar films. In recent years, some studies have pointed out that the quality of polar thin films can be significantly improved by high-temperature annealing (HTA) technology [20]. Unfortunately, there are still few studies on the annealing of semi-polar and non-polar thin films with great application potential. In this work, thin AlN films were deposited on a-, m- and r-sapphire substrates and HTA was used to improve the crystal quality of the films. The influence of HTA on AlN films with different polarities was studied and the different influences were explained by the annihilation of impurities and grain boundaries during the annealing process.

## 2. Materials and Methods

**Figure 1:**
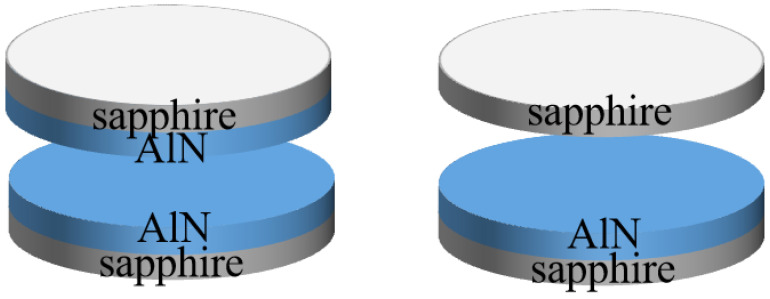
Schematic diagram of face-to-face setup.

Polar (0001), semi-polar (11−22) and non-polar (11−20) AlN films were deposited on a-, m- and r-sapphire substrates, respectively, by magnetron sputtering at 650 °C and 700 W. Oxygen, nitrogen and argon were employed for the plasma that reacts to aluminum target. In the initial stage, a small flux of O_2_ was introduced to form a very thin AlON layer (about several nanometers) on the substrate to prepare high-quality AlN films. The thickness of all sputtered AlN films was 300 nm. The samples were treated with HTA under 150 Torr in a nitrogen atmosphere at 1400, 1500, 1600 and 1650 °C for 30 min. To inhibit the thermal decomposition of the AlN film during thermal annealing, the surface of the AlN film was covered with another sample or sapphire in a face-to-face setup, as shown in Figure 1.

The crystallinity of the AlN films was measured by high-resolution X-ray diffraction (XRD). The XRD results were obtained by using a PANalytical instrument with a Cu K_α1_ = 1.5406 Å radiation. Raman spectroscopy, scanning electron microscopy (SEM) and X-ray photoelectron spectroscopy (XPS) were used to further characterize the quality, stress and surface morphology of the films. Raman spectra were obtained using a Labram HR Evolution instrument with a laser of wavelength 532 nm. SEM images were obtained using a VEECO Dimension 3100 instrument. XPS was performed using an ESCALAB 250XI+ instrument with a monochromatic Al target X-ray source.

## 3. Results and Discussion

**Figure 2:**
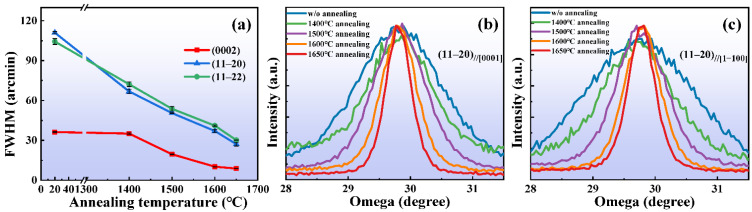
(**a**) The FWHM of rocking curves of polar (0002), semi-polar (11–22) and non-polar (11–20) films after annealing at 1400–1650 °C as a function of annealing temperature. The XRC of the non-polar (11–20) film along (**b**) [0001] direction and (**c**) [1−100] direction without and with annealing.

Firstly, we investigated the effect of annealing temperature on film quality with XRD. Figure 2a shows the relationship between the full-width at half-maximum (FWHM) of the XRD rocking curves (XRC) of the polar, semi-polar and non-polar films, and the annealing temperature. With the increase in annealing temperature, the FWHM of the films decreased and the film quality was improved. However, there was still a certain gap between the quality of the semi-polar and non-polar films and that of the polar film after HTA. The polar films exhibited the highest quality after annealing. Additionally, the quality of the non-polar film was better than that of the semi-polar film after HTA, which may be related to the content of impurities in the films before and after annealing.

The non-polar thin film had structural anisotropy due to the asymmetry of the crystals in the plane. The anisotropy of the thin-film structure was caused by the anisotropy of atomic diffusion length or growth rate on the growth surface [21]. Figure 2b,c show XRC from two different incident beam directions, the c-axis [0001] and the m-axis [1−100] of AlN. It can be seen that the rocking curve of the non-polar film was wide before annealing, which indicated that the sputtered film had a high density of dislocation. As the annealing temperature was increased, the rocking curves of the non-polar films became smooth and sharp, and the FWHM decreased, which indicated that HTA significantly reduced the dislocations in the films. Though the crystal quality along the c-axis and the m-axis was improved, the FWHM values of the XRCs in the two directions were different. The FWHM along the c-axis [0001] direction was smaller than that in the [1−100] direction, which was mainly attributed to the smaller stacking fault density and the faster growth rate along the c-axis [0001]. Due to the difference in the in-plane growth rates, the crystal mosaic degree also exhibited anisotropy. With the increase in annealing temperature, the difference in FWHM between the two perpendicular directions decreased, and the anisotropy of the films decreased gradually, as shown in Table 1. The anisotropy was related to the stacking fault density in the film; HTA resulted in the decrease in the growth rate difference between the two directions, which reduced the stacking fault density and improved the film quality.

**Figure 3:**
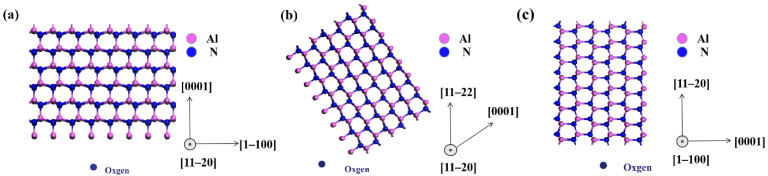
Impurity-binding model for (**a**) polar (0001), (**b**) semi-polar (11–22) and (**c**) non-polar (11–20) films.

The formation of dislocations was often related to impurities. We carried out high-resolution XPS to analyze the changes in the impurity concentration in the films. The Al 2p, N 1s, O 1s and C 1s in the films were scanned with high resolution to analyze atomic concentrations (at. %). The surface element concentrations were calculated according to the element characteristic peak area and the corresponding element sensitivity factor [22]. The element concentrations of the unannealed samples are shown in Table 2. On account of AlN exhibiting a high affinity for oxygen, oxygen atoms easily entered the lattice to form defects [23]. When oxygen atoms entered the AlN lattice, oxygen replaced nitrogen atoms in the lattice to form aluminum vacancies [24]. As the oxygen content increased, the defects agglomerated and evolved into extended defects [24,25]. The impurity model was used to explain the difference in the oxygen impurity levels and the quality of the films with different polarities (shown in Figure 3). It can be seen that when an oxygen atom tried to enter the lattice of the AlN film, the aluminum atom of the polar structure film prevented the oxygen impurity from entering the lattice to replace the nitrogen atom. The structure of the semi-polar film was inclined at a certain angle compared with the polar film, which weakened the blocking effect of the aluminum atoms on oxygen contamination. In contrast, the non-polar film exhibited no blocking effect of aluminum atoms on impurities; therefore, non-polar films may contain more oxygen impurities and defects. Moreover, this was consistent with the XPS test results (as shown in Table 2), which showed that the non-polar film was more vulnerable to impurities than the polar and semi-polar films. This may be the reason why the quality of the non-polar film was lower than that of the polar and semi-polar films before annealing.

**Figure 4:**
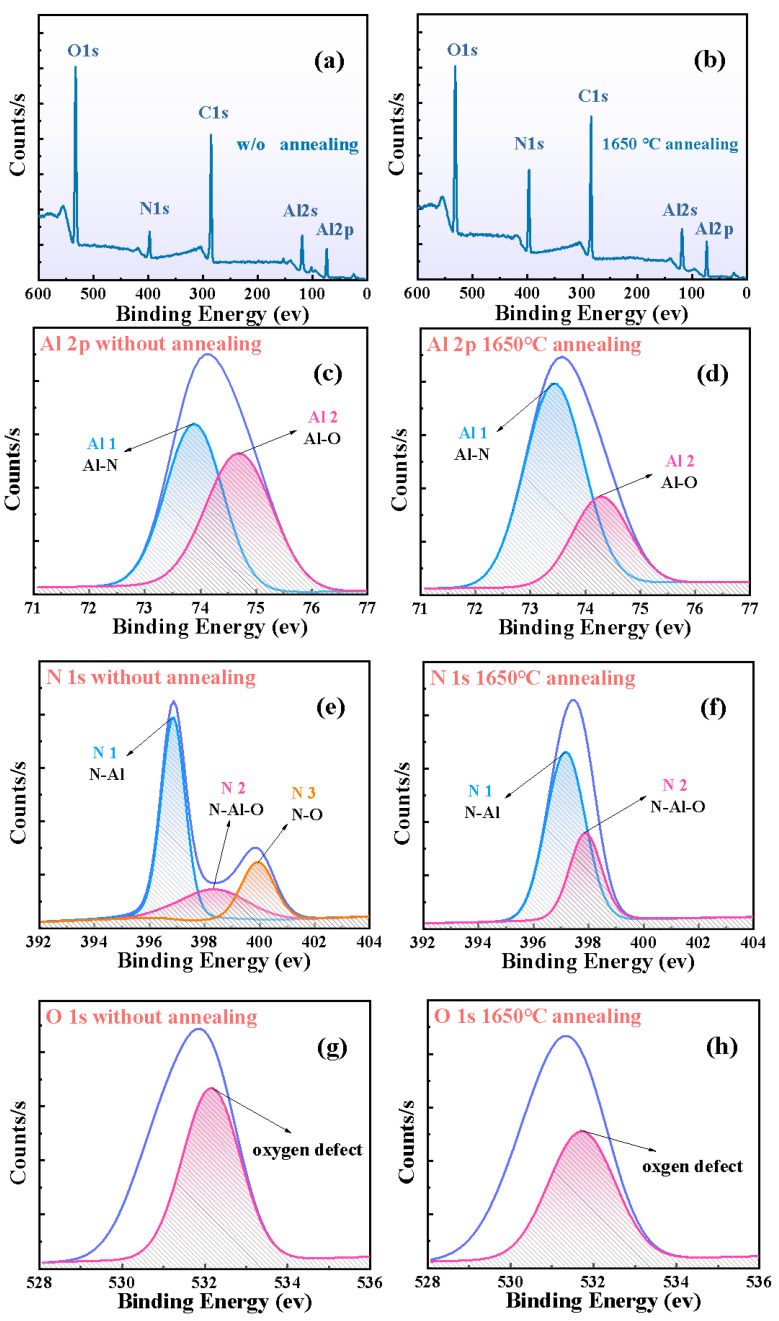
The XPS high-resolution scans for the non-polar film (**a**) without annealing and (**b**) with annealing at 1650 °C. The subpeak spectra of (**c**) Al 2p, (**e**) N 1s and (**g**) O 1s peaks on the surface of the non-polar film without annealing, and (**d**) Al 2p, (**f**) N 1s and (**h**) O 1s peaks with annealing at 1650 °C.

In order to further investigate the micromechanism of the quality improvement of the non-polar film after annealing, high-resolution XPS tests were carried out. Figure 4 shows the XPS results of non-polar films with and without annealing. The concentrations of O and C impurities in the films after thermal annealing changed to 17.64% and 40.85%, respectively, and those of Al and N increased to 24.29% and 17.22%, respectively (Figure 4a,b). This means that HTA reduced the concentration of impurities in the non-polar film. A linear combination of Gaussian and Lorentz functions was used to fit the subpeaks of Al 2p and N 1s. The subpeaks within each element peak represented different chemical states. The phase information of non-polar films can be obtained by analyzing the area ratio of subpeaks during annealing. The Al 2p peak contained two subpeaks, A 1 and A 2, which appeared at 73.5 eV and 74.7 eV, respectively (shown in Figure 4c) [26]. The A1 subpeak originated from the Al-N bond in wurtzite and existed in the form of aluminum nitride phase [22]. The A 2 subpeak was derived from the Al-O bond and could be attributed to the alumina domains and the oxidation of AlN grain boundaries [22]. The proportion of Al-N and Al-O bonds changed from 46.7% and 46.1% for the as-grown sample to 66.4% and 27.3% after annealing at 1650 °C, respectively. These results suggested that HTA enhanced the Al-N interaction in the non-polar film and strengthened the AlN phase. The N 1s peak of the non-polar film before and after annealing contained two subpeaks, N 1 and N 2, which were related to the interaction between the N and Al atoms (shown in Figure 4e). In the as-grown non-polar film, in addition to N 1 and N 2 subpeaks, the N 1s peak also had a subpeak at higher binding energies (marked as N 3). This subpeak was the result of the interaction between nitrogen and oxygen atoms on the film surface [22]. The subpeak N 3 disappeared and the areas of the N 1 and N 2 subpeaks increased after HTA (as shown in Figure 4e,f), which meant the N-O bond broke and the interaction between N and Al increased. Therefore, the crystallinity of the film improved.

Table 3 shows the concentrations of different atoms in all the samples after annealing at 1650 °C. After annealing at high temperature, the concentration of oxygen impurities in the non-polar film decreased from 29.61% to 17.64%, while that in polar film decreased from 26.03% to 14.49% and in the semi-polar film decreased from 28.96% to 23.81%. This indicated that the non-polar film was strongly affected by HTA and the impurity concentration was greatly reduced. The aggregation of screw dislocations was conducive to the adsorption of oxygen impurities [27]. Consequently, the reduction in the oxygen impurity concentration proved that HTA effectively reduced the dislocations in the films, such that the quality of the non-polar film after annealing was higher than that of the semi-polar film, which was consistent with the XRC tests. Through the fitting of the O 1s spectra, a subpeak was obtained near 531.8 eV, which originated from the Al-O octahedral complex in the grain and was related to oxygen defects [24,26]. After HTA, the proportion of this subpeak decreased from 53.4% to 42.6%. This should be assigned to HTA, which reduced the concentration of oxygen impurities, inhibited the formation of defects, repaired the lattice damage of the film and improved the quality of the non-polar film.

**Figure 5:**
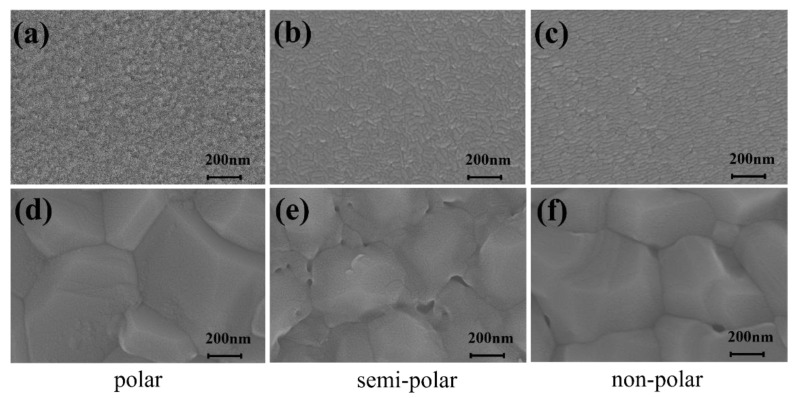
(**a**–**c**) The surface morphology images of the films without annealing; (**d**–**f**) the surface morphology images of the films with annealing at 1650 °C.

The microstructure of the AlN films was characterized by SEM to clarify the mechanism of HTA of the films, as shown in Figure 5. Before annealing, the surfaces of the films were covered with a large number of small particles, which indicated that the films deposited by sputtering were uniform. In addition, the surfaces of the films showed discrete grains and a large number of grain boundaries, showing that the film had a high density of dislocation. The surface grains of the semi-polar and non-polar films were tilted long strips. This was due to anisotropic growth resulting in thin-film stripe characteristics. After annealing at high temperature, the grains of the polar, semi-polar and non-polar films increased obviously and the grain boundaries decreased. The grain boundary contained dislocations, impurity atoms and other defects. This indicated that the recrystallization process reduced the grain boundaries of the films, thus reducing the defects and improving the crystal quality of the films. The surfaces of the semi-polar and non-polar films after annealing no longer exhibited obvious grain characteristics of long stripes. The crystal quality of the non-polar film after annealing was still a degree lower than that of the polar film. However, compared with the semi-polar film, the non-polar film had a higher crystal quality with a larger grain size and fewer holes on the surface. This confirmed that the anisotropy properties of the semi-polar and non-polar films were reduced after HTA, and that the quality of the non-polar film could be improved more easily through thermal annealing than that of the semi-polar film.

**Figure 6:**
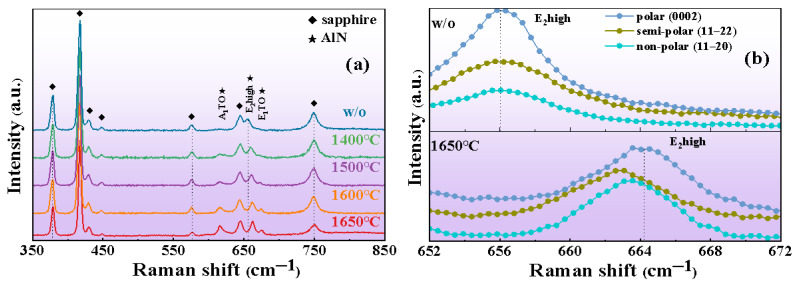
(**a**) Raman spectra of the non-polar film without and with annealing. (**b**) Raman spectra of polar, semi-polar and non-polar films without and with annealing at 1650 °C.

Raman spectra were used to analyze the effect of HTA on the stress state and quality of the films. For an AlN crystal, the frequency shift of the E_2_ (high) phonon peak position is generally used to reflect the stress of the AlN film. The E_2_ (high) peak in stress-free bulk AlN was 657.4 cm^−1^ [28,29]. The E_2_ (high) peak of the sputtered AlN films was located at 656.0 cm^−1^, which indicated that the stress state of the sputtered film was tensile stress. After annealing at high temperature, the E_2_ (high) Raman peak of the non-polar film was blue shifted, as shown in Figure 6a. The blue shift increased with the increase in annealing temperature and the shifted peak was greater than 657.4 cm^−1^. This phenomenon showed that after annealing, the stress of the film was released, and the film changed from tensile stress to compressive stress. The change of stress state may be attributed to the recrystallization process in the film during annealing. In the recrystallization process, there was lattice mismatch and thermal-expansion mismatch between the epitaxial AlN film and the sapphire substrate, which led to compressive strain [30]. The dislocation contributed to the relaxation of the stress in the film [31]. The compressive stress inside the AlN films gradually increased with the enhanced annealing temperature, proving that annealing reduced the dislocation in the film and weakened the relaxation effect of dislocation. Raman peak linewidth was used as a measure of phonon lifetime [32]. When the film contained a large number of grain boundaries and other defects, phonons will decay at grain boundaries or defects during the Raman scattering process, resulting in a shortened lifetime and a widening of Raman linewidth. Thus, the film quality could be obtained from the variation of the linewidth of the Raman phonon peak. It can be seen from the Raman spectra that the linewidth of the E_2_ (high) peak decreased gradually with the increase in annealing temperature. In addition to the E_2_ (high) phonon modes, A_1_ (TO) and E_1_ (TO) modes were observed in our Raman spectra. Simultaneously with the increase in annealing temperature, the FWHM of the A_1_ (TO) and E_1_ (TO) modes also decreased. The atomic vibrations of the A_1_ (TO) and E_1_ (TO) modes were along the c-axis and m-axis, respectively [33]. This proved that the crystal quality of the non-polar film along the c-axis and the m-axis was improved after HTA. In addition, Figure 6b illustrates that at the same annealing temperature, the E_2_ (high) peak offset of films with different polarities was different and the stress of the films was different. This may be due to different degrees of dislocation reduction in the recrystallization process of the films with different polarities, resulting in the different stress and crystallization qualities of the films.

## 4. Conclusions

In summary, the thermal annealing of AlN films with different polarities was studied in this paper. The quality of polar, semi-polar and non-polar films was improved by thermal annealing. The films with different polarities had different degrees of quality improvement. Although there was a gap between the quality of the non-polar film and that of the polar film after annealing, compared with the semi-polar film, the non-polar film was easier to treat to achieve quality improvement. XPS tests showed that the quality difference between the films was related to the concentration of impurities. Compared with the semi-polar film, the concentration of impurities in the non-polar film was greatly reduced. The thermal annealing process could effectively reduce impurities and inhibit the formation of defects in the film, and the non-polar film was greatly affected by the annealing temperature. Combined with the surface morphology images, we can draw the conclusion that under the treatment conditions used in this study, the essence of crystal quality improvement lies in the annihilation of grain boundaries during the annealing process. In addition, HTA reduced the anisotropy of the non-polar and semi-polar films, improving the quality of these films. The blue shift of the Raman phonon peak and the reduction in the linewidth further revealed that the stress state and quality of the films changed during annealing. The films with different polarities exhibited different stresses after HTA. During the annealing process, the stress state of the films changed from tensile stress to compressive stress, the dislocation and defects of the films were reduced and the quality of the films improved.

## Figures and Tables

**Table 1:** The FWHM (arcmin) of non-polar film along two perpendicular directions without and with annealing.

Direction	W/O	1400 °C HTA	1500 °C HTA	1600 °C HTA	1650 °C HTA
[0001]	111	73	57	37	26
[1−100]	159	87	60	38	27

**Table 2:** The elemental concentrations of the unannealed polar, semi-polar and non-polar films.

Sample	Al (at. %)	N (at. %)	C (at. %)	O (at. %)
Polar	15.68	8.41	49.88	26.03
Semi-polar	15.62	5.49	49.93	28.96
Non-polar	15.43	4.93	50.03	29.61

**Table 3:** The elemental concentrations of the polar, semi-polar and non-polar films with annealing at 1650 °C.

Sample	Al (at. %)	N (at. %)	C (at. %)	O (at. %)
Polar	25.29	20.03	40.19	14.49
Semi-polar	16.73	17.19	42.37	23.81
Non-polar	24.29	17.22	40.85	17.64

## Data Availability

Data sharing is not applicable to this article.
